# How can we possibly resolve the planet's nitrogen dilemma?

**DOI:** 10.1111/1751-7915.14159

**Published:** 2022-11-15

**Authors:** Silvio Matassa, Pascal Boeckx, Jos Boere, Jan Willem Erisman, Miao Guo, Raffaele Manzo, Francis Meerburg, Stefano Papirio, Ilje Pikaar, Korneel Rabaey, Diederik Rousseau, Jerald Schnoor, Peter Smith, Erik Smolders, Stefan Wuertz, Willy Verstraete

**Affiliations:** ^1^ Department of Civil, Architectural and Environmental Engineering University of Naples Federico II Naples Italy; ^2^ Department of Green Chemistry and Technology, Faculty of Bioscience Engineering Ghent University Ghent Belgium; ^3^ Allied Waters B.V. Nieuwegein The Netherlands; ^4^ Institute of Environmental Sciences Leiden University Leiden The Netherlands; ^5^ Department of Engineering, Faculty of Natural, Mathematical and Engineering Sciences King's College London London UK; ^6^ Aquafin NV Aartselaar Belgium; ^7^ School of Civil Engineering The University of Queensland Brisbane Queensland Australia; ^8^ Center for Microbial Ecology and Technology (CMET), Faculty of Bioscience Engineering Ghent University Ghent Belgium; ^9^ Department of Civil and Environmental Engineering University of Iowa Iowa City Iowa USA; ^10^ Institute of Biological and Environmental Sciences University of Aberdeen Aberdeen UK; ^11^ Division Soil and Water Management Katholieke Universiteit Leuven Leuven Belgium; ^12^ Singapore Centre for Environmental Life Sciences Engineering (SCELSE), Nanyang Technological University Singapore Singapore; ^13^ School of Civil and Environmental Engineering, Nanyang Technological University Singapore Singapore

## Abstract

Nitrogen is the most crucial element in the production of nutritious feeds and foods. The production of reactive nitrogen by means of fossil fuel has thus far been able to guarantee the protein supply for the world population. Yet, the production and massive use of fertilizer nitrogen constitute a major threat in terms of environmental health and sustainability. It is crucial to promote consumer acceptance and awareness towards proteins produced by highly effective microorganisms, and their potential to replace proteins obtained with poor nitrogen efficiencies from plants and animals. The fact that reactive fertilizer nitrogen, produced by the Haber Bosch process, consumes a significant amount of fossil fuel worldwide is of concern. Moreover, recently, the prices of fossil fuels have increased the cost of reactive nitrogen by a factor of 3 to 5 times, while international policies are fostering the transition towards a more sustainable agro‐ecology by reducing mineral fertilizers inputs and increasing organic farming. The combination of these pressures and challenges opens opportunities to use the reactive nitrogen nutrient more carefully. Time has come to effectively recover used nitrogen from secondary resources and to upgrade it to a legal status of fertilizer. Organic nitrogen is a slow‐release fertilizer, it has a factor of 2.5 or higher economic value per unit nitrogen as fertilizer and thus adequate technologies to produce it, for instance by implementing photobiological processes, are promising. Finally, it appears wise to start the integration in our overall feed and food supply chains of the exceptional potential of biological nitrogen fixation. Nitrogen produced by the nitrogenase enzyme, either in the soil or in novel biotechnology reactor systems, deserves to have a ‘renaissance’ in the context of planetary governance in general and the increasing number of people who desire to be fed in a sustainable way in particular.

## Graphical abstract

Nitrogen is the most crucial element in the production of nutritious feeds and foods, yet, the production and massive use of fertilizer nitrogen constitutes a major threat in terms of environmental health and sustainability. Time has come to implement a more sustainable and circular management of nitrogen by recovering used nitrogen from secondary resources and by leveraging the exceptional potential of biological nitrogen fixation.

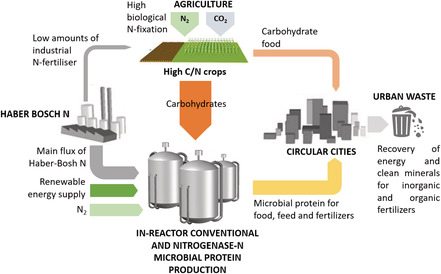

## NITROGEN AS A NECESSITY BUT ALSO A THREAT

People dealing with sanitation know it: per person per day, some 13–14 g of ‘used nitrogen’ is excreted and needs, at present by the best available technology, to be removed ‐ that means reconverted from the active form to the dinitrogen form. Hence, every person needs per day via their food uptake about 13–14 g of nitrogen, in the form of nutritious proteins, to maintain good health and functionality (Courtney‐Martin & Pencharz, 2016). Through photosynthesis, food plants produce proteins, but to do this they require an ample supply of nitrogen. Most plants require the so‐called reactive nitrogen as source of nitrogen, that is, nitrogen in the form of ammonia, nitrate, urea or amino acids. Some plants can fix nitrogen, but they do this with the help of microorganisms. The nitrogenase enzyme, which is capable of biologically fixing non‐reactive dinitrogen gas (which constitutes 78% of the air) into reactive N, is only produced by microscopic forms of life, that is, bacteria such as Rhizobia and Azotobacter (Robson et al., 2015) and many other cyanobacteria (Stal, 2015). It was quite understandable that around 1798 the British philosopher Thomas Malthus, when observing the rapid growth of the human population, and hence the need for proteinaceous food, predicted a dark future for mankind because in those days, all conversion of atmospheric nitrogen to a form of reactive nitrogen that could be used in the food chain, relied on biological nitrogen fixation (BNF) (Gilland, 1993). And in those times, these processes were not understood at all, and famine was constantly present in the world.

With the turn of the 20th century, a ‘miraculous’ game change came. Around 1909, while searching to expand the production of gunpowder (consisting of sulphur, carbon and nitrate nitrogen), some of the top chemists of Germany, Fritz Haber and Carl Bosch, succeeded in producing reactive nitrogen from dinitrogen gas in the air. They were developing better weaponry, and by accident invented chemical fertilizers. Since then, by using fossil fuel to provide hydrogen and assure high temperatures and pressures, atmospheric dinitrogen gas is activated and converted to form ammonia. Such reactive nitrogen is then used for many purposes, including explosives, fibres, plastics and pharmaceuticals, but particularly to provide farmers with the chemicals required to supply plants with the essential reactive nitrogen to grow abundantly. Therefore, through artificial fertilizers production, the fossil fuel‐based industry has been a cornerstone of our protein supply (Smil, 2004) and currently provides more than 50% of the proteins in our food (Erisman et al., 2008).

As a result, a century after this serendipitous discovery, we have turned to massive production and use of reactive nitrogen on our planet. We produce some 100 million tons of nitrogen fertilizer per year (Matassa, Batstone, et al., 2015; Matassa, Boon, & Verstraete et al., 2015). This corresponds to about 13 kg of fertilizer nitrogen per person per year globally, which is not distributed equally over the world. The farmer needs for a crop such as wheat or potato, some 100 kg nitrogen per ha to meet the nitrogen requirements of the plant. Yet, due to the relatively low cost of synthetic fertilizers, there is a tendency to add double to triple the amount that is needed (O'Beirne & Cassidy, 1990). Because of the large availability of cheap synthetic fertilizers, the long‐established and balanced manure‐crop system was decoupled, and artificial N is now the main nutrient source used to produce feed for animals. Currently, about 50% of habitable land is devoted to agricultural feed and food production, while almost 80% of the latter agricultural land is used to sustain livestock production (Our World in Data, 2022). As a matter of fact, the number of domestic animals have increased enormously together with the generation of excess manure, which contributes to overfertilization, leading to losses to the atmosphere and water, causing pollution, contributing to climate change, biodiversity loss, etc. Such systematic overfertilization and poor nitrogen management have led to a dramatic decline of the so‐called nitrogen use efficiency (NUE), which currently ranges around 50% for the majority of conventional crops and cultivation systems (Ladha et al., 2016). As indicated, technically, we need some 13–14 g per person per day, which on averagely corresponds to 5 kg nitrogen per person per year. Yet, to obtain this in the form of proteinaceous food on the table, considering this low NUE and losses in the food chain, we actually depend on an average of 13 kg reactive nitrogen fertilizers per person per year, thus almost three times higher than what is strictly needed. At present, we have optimized the process so that only 2 L fossil fuel, which is equivalent to about 40 MJ, are needed to produce 1 kg of reactive nitrogen, corresponding with a fossil fuel equivalent of some 100 L per person per year worldwide for nitrogen fertilizer supply (see also Box 1). Thus, the overall fossil fuel used to produce mineral fertilizer represents a significant 1 to 2% of all fossil fuel consumption, accounting for about 1.4% of the global CO_2_ emissions (Kyriakou et al., 2020).

Box 1 **Approximative stoichiometric and volumetric considerations**

The following considerations aim at comparing the energy efficiency of the various nitrogen fixation routes with the minimum energy required to convert dinitrogen gas into reactive ammonia through hydrogen gas (Equation (1)).

(1) $$N_{2}+3H_{2}\to 2{\mathrm{NH}}_{3}$$

According to Equation (1), in plain chemical equivalents, 6 kg of hydrogen gas generates 28 kg of ammonia nitrogen. By further considering that each kilogram of hydrogen has a primary energy content of 120 MJ, the 6 kg of hydrogen required to fix 28 kg of reactive ammonia nitrogen, is equivalent to 25.7 MJ/kg N.

Table 1 reports the amounts of primary energy required to fix 1 kg of nitrogen from air and the corresponding volumetric productivities (i.e. kg of N fixed per m3 bio‐reactive system per day).

**TABLE 1:** Energetic efficiency and volumetric productivity of artificial and biological nitrogen fixation routes

N‐fixing process	Primary energy requirement (MJ/kg N)	Energetic efficiency (%)^a^	Volumetric productivity (kg N/m^3^·d)
Haber–Bosch	40^b^	64.3	120^c^
Biological N‐fixation in the root‐nodule‐rhizobia	200^d^	12.9	0.75^e^
Biological N‐fixation through free living aerobic bacteria	790^f^	3.2	0.02^g^
Biological N‐fixation through free living anaerobic bacteria	1580^h^	1.6	0.1^i^

^a^ Calculated by dividing the primary energy requirement by the minimum energy requirement of 25.7 MJ/kg N.

^b^ Matassa, Batstone, et al. (2015), Matassa, Boon, and Verstraete (2015).

^c^ Smith et al. (2020).

^d^ Smil (2004).

^e^ By assuming 1000 kg of fresh weight nodule per hectare and 75 kg N fixed per 100 days of growth season, one arrives at a rate of 0.75 kg N fixed per m^3^ reactive fresh biomass per day.

^f^ Calculated by considering a requirement of 50 kg of carbohydrates per kg N fixed (Din et al., 2019) and an energy density of 15.8 MJ/kg carbohydrate consumed (Aniza et al., 2021).

^g^ Din et al. (2019).

^h^ Calculated by considering a requirement of 100 kg of carbohydrates per kg N fixed (Din et al., 2019) and an energy density of 15.8 MJ/kg carbohydrate consumed (Aniza et al., 2021).

^i^ Calculated by considering the volume of termite gut where anaerobic N fixation occurs (Lilburn et al., 2001).

In recent decades, it has become clear that decreasing the use of mineral nitrogen fertilizers could help to curb fossil fuel use and CO_2_ emissions. And there is more. The prices of mineral fertilizer were thus far quite low, around 0.70 Euros per kg nitrogen in 2015 (Matassa, Batstone, et al., 2015; Matassa, Boon, & Verstraete, 2015). This allowed considerable amounts of nitrogen to be used in agriculture, with little interest in enhancing nitrogen use efficiency or nitrogen recovery. The reactive nitrogen not used by the plant then becomes a burden to the environment. First of all, it washes out from the fields as nitrate and enriches the waterways and water bodies, where it gives rise to eutrophication. Secondly, it accumulates in surface and groundwater and thus can reach levels above the 10 mg nitrate nitrogen per litre, which are considered to be potentially harmful to the health of the consumer (Ward et al., 2018). Moreover, in soil and sediment environments which are low in oxygen, nitrate can be converted by microorganisms to nitrous oxide (also called laughing gas, N_2_O). The nitrous oxide migrates to the stratosphere and destroys the protective ozone layer, and also has an about 300 times stronger impact on global warming than CO_2_ on a 100‐year timescale (Griffis et al., 2017).

In addition, the intensive use of nitrogen fertilizer results in massive emissions of reactive nitrogen (particularly NOx and ammonia) in the air, particularly via the intensive husbandry of animals such as pigs, chickens and cattle. Clean air contains some 0.1 μg ammonia nitrogen per cubic meter; air ventilated from concentrated animal feeding operations (CAFOs) such as hog confinements contains some 1000 μg ammonia per cubic meter (Preece et al., 2017). Clearly, industrial animal rearing units can emit several thousands of kg of ammonia per month. This subsequently leads to ammonia being washed from the air and deposited in natural areas at levels of up to 12–14 kg of reactive N per ha per year (Tanner et al., 2022). This deposition of reactive nitrogen strongly disturbs the ecology of plant species, and concomitantly of insects and higher animals, in these normally nutrient‐poor ecosystems. Fast‐growing plants become dominant, for example, grasses and brambles, and eutrophication occurs across the landscape. Overall, the latter results in a major threat to the biodiversity and thus to the health of natural ecosystems, and the ecosystem services they provide (Horswill et al., 2008). Clearly, the excessive use of nitrogen fertilizers constitutes a variety of threats to our planet (Gretchen & Matson, 2008).

**FIGURE 1:**
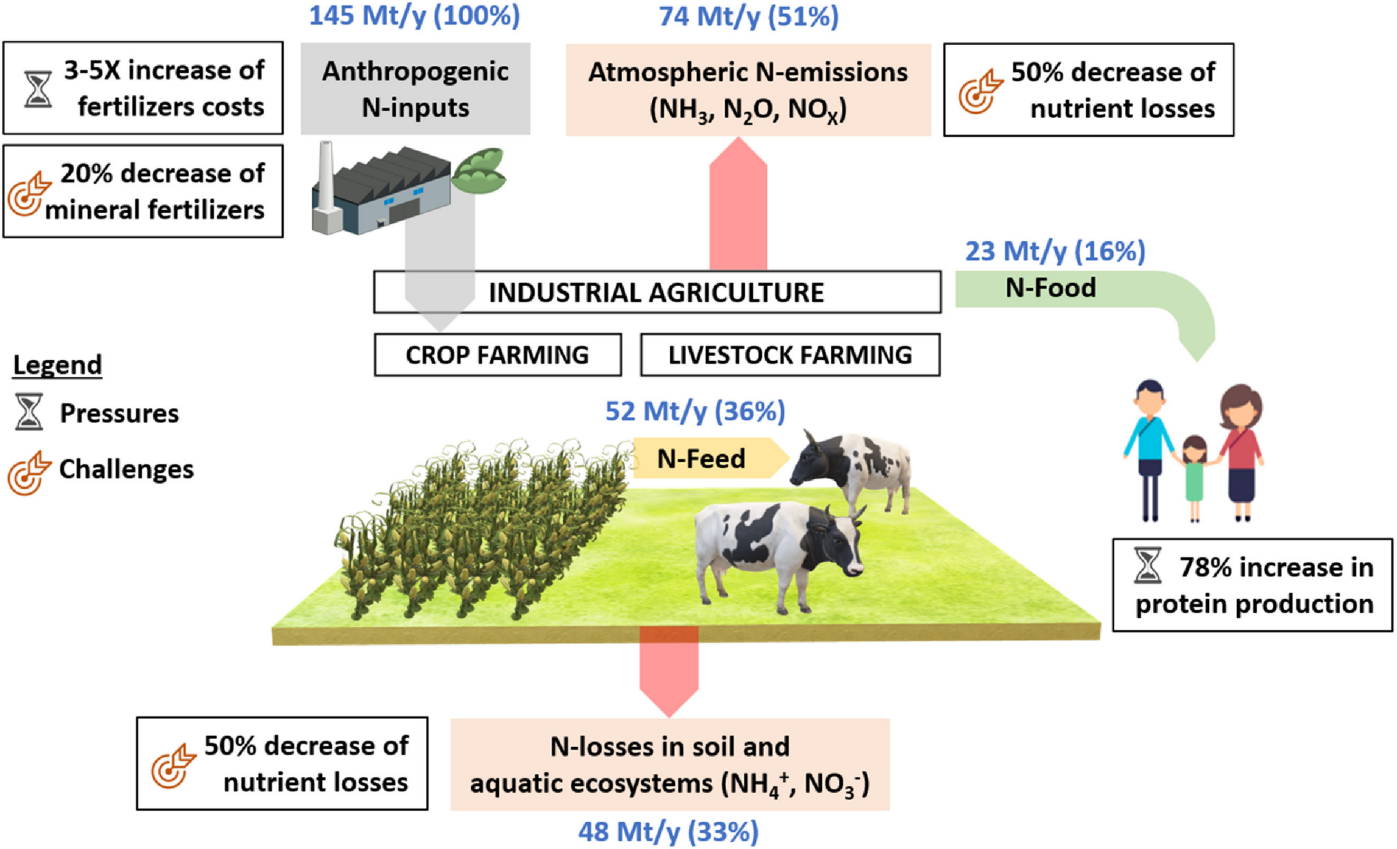
Overview of the impact of the anthropogenic nitrogen cycle on the biosphere together with some of the main pressures and challenges arising from international policies and socio‐economic dynamics. The annual anthropogenic N‐flows (text in blue), taken from Matassa, Batstone, et al. (2015), Matassa, Boon, and Verstraete (2015), consider the Haber–Bosch process (100 Mt), biological fixation in crops (35 Mt) and deposition in animal rearing (10 Mt) for the N‐inputs. Volatilization from the field (48 Mt) and loss/volatilization from manure storage (26 Mt) for atmospheric N‐emissions. Vegetable (13 Mt) and animal (10 Mt) protein sources for the N‐food. The ‘challenges’ relate to the targets set out in the European green Deal's farm to fork strategy and biodiversity strategy (Montanarella & Panagos, 2021), while the ‘challenges’ relate to the current rise in fertilizers production cost (Behnassi & el Haiba, 2022) as well as the projected increase in protein production required to meet global demand by 2050 (Henchion et al., 2017).

In countries such as the Netherlands and Belgium, the problems of excessive nitrogen in the environment are of extreme political priority and dominate a number of economic investments and policy considerations (Klages et al., 2020). Indeed, the current excessive use of nitrogen is considered as one of the most urgent matters in relation to the sustainable planetary boundaries of the Earth system (Rockstrom et al., 2009; Steffen et al., 2015). We should not only talk about CO_2_, but also about reactive nitrogen, in terms of the sustainability of our planet. A radical new approach is needed to curb anthropogenic nitrogen pollution and create a circular economy for nitrogen (Figure 1).

## STRAIGHTFORWARD SOLUTIONS TO THE ACUTE NITROGEN PROBLEM

**FIGURE 2:**
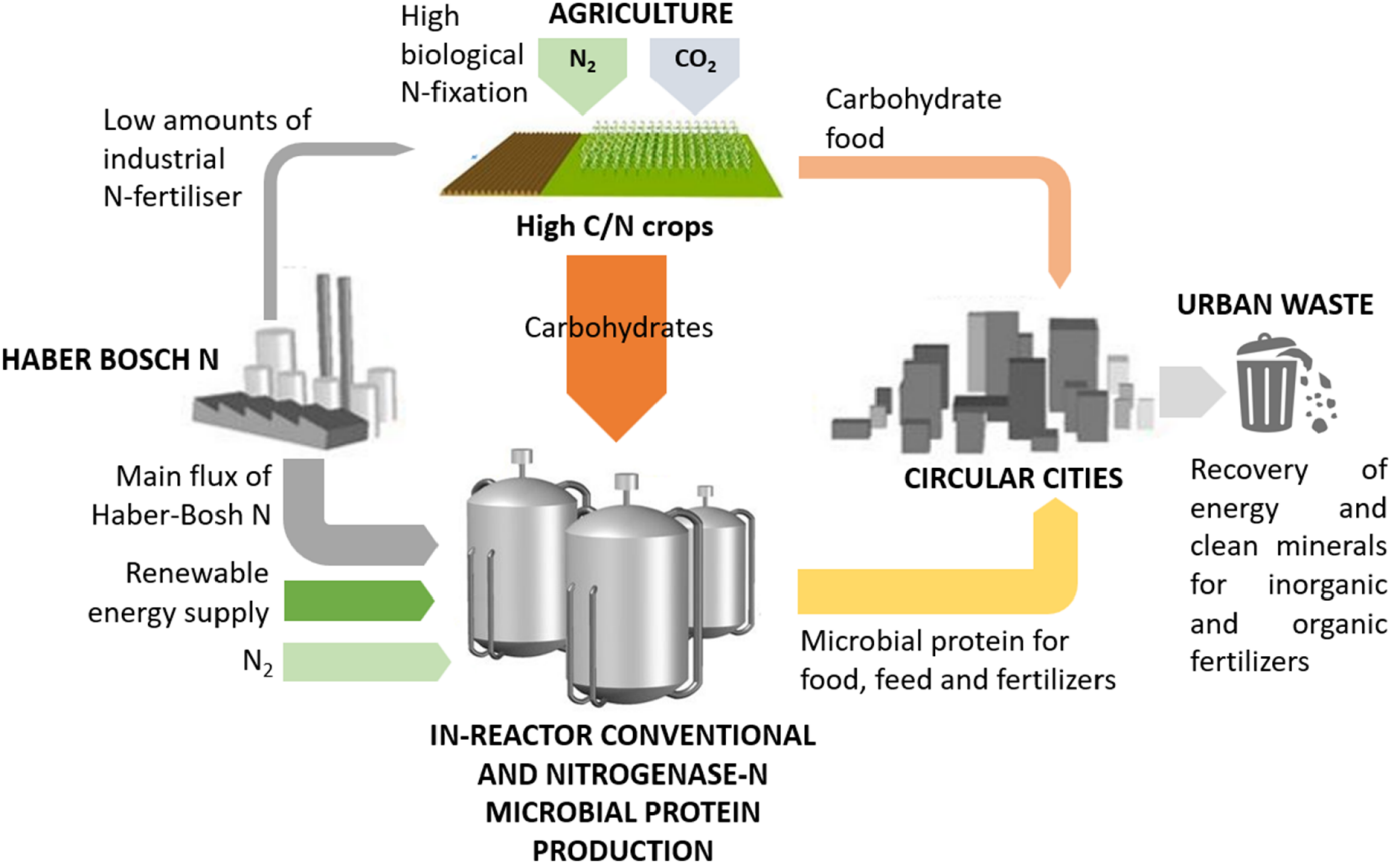
Scheme of biorefinery upgrading of high carbon to nitrogen (C/N) crops to microbial protein biomass, which can serve as a protein‐rich food, feed or organic fertilizer. By using such a biotechnological concept for protein production, considerable amounts of conventional agricultural land can be made available for other purposes (Pikaar et al., 2018; Pikaar et al., 2018).

Of course, the consumption of animal products such as meat and dairy are paramount driving forces in the global nitrogen use pattern. It is evident that the shift in the overall consumer behaviour towards patterns not involving the so‐called production animal is needed in order to substantially alleviate the current nitrogen stress on the planet. Indeed, most crops achieve nitrogen uptake efficiencies of only 50% or less of what is provided, and animals convert at the very best only 30% of what they consume to high‐value products (Congreves et al., 2021; Godinot et al., 2015). Microorganisms growing directly on carbohydrates and mineral nitrogen (as depicted in Figure 2) can have efficiencies of N uptake which are of the order of 100% (Pikaar et al., 2017; Upcraft et al., 2021). The next best approach is to use reactive nitrogen more efficiently. Precision farming allows fertilizers, for instance by geo‐positioned control of fertilizing machinery, in the exact square meter where a given amount of plant nutrient is needed (Klages et al., 2020). This well‐designed dosage should be combined with intensive and controlled release measures such as monitoring the amount of residual nitrogen in the soil, in the groundwater and in the surrounding surface waters. Replacing mineral nitrogen fertilizer with organic nitrogen, in the form of protein‐rich biomass such as microbial cells, is possible. Microbial protein has indeed shown to mineralize very fast in soil, having equal N fertilization potential as regular organic fertilizer and potentially delivering other additional benefits to crops (e.g. biostimulation and biofortification) (Sakarika et al., 2020; Spanoghe et al., 2020). The latter is directly reflected in the market price of organic nitrogen, which can attain a 2 to 3 higher market value than that of mineral Haber–Bosch nitrogen (consider 5–6 against the current 2 Euro per kg N respectively) (Hartz & Johnstone, 2006). Since the onset of the 2022 war in Ukraine, a new set of considerations have gained attention. Indeed, due to the rising prices of fossil fuel, the prices of mineral nitrogen also rose strongly. Actually, they have increased at an unprecedented (factor 3–5) rate. At present, the world market price of N is of the order of 2–4 Euros per kg nitrogen (Behnassi & el Haiba, 2022). This has never previously been seen and will certainly contribute to a much more careful use of mineral fertilizer all over the world, as already prioritized by international policies such as the European Green Deal, targeting a 20% lower mineral fertilizer use (Montanarella & Panagos, 2021). Hence, the potentials offered by microbial nitrogen fertilizers must be reconsidered, and future research must be devoted to tailor their composition to guarantee high fertilizing efficiencies under different agronomic conditions.

### Microbially catalysed protein production

A subsequent line of action is to develop other forms of protein, not relying on the use of nitrogen fertilizers in open fields which unavoidably leads to high N losses due to factors such as drought, rain, weeds and undesirable microbial processes (Matassa, Batstone, et al., 2015; Matassa, Boon, & Verstraete, 2015). This is particularly of concern when crops are grown for protein production such as fodder grasses, wheat and soy. By shifting from mineral N‐intensive plant protein production to the cultivation low N‐demanding plants that are characterized mainly by carbohydrate production, one can ‘tap the sunlight’ in the field to synthesize plant carbohydrates such as sugar, starch and cellulose. Sugarcane, potato and tapioca, grasses for fibre and trees are typical examples of such carbohydrate‐rich crops. The carbohydrates thus produced in the field from sunlight, can be brought to the biorefinery and supplemented with reactive nitrogen (obtained through Haber–Bosch but also via nitrogenase enzymes—see further), where under controlled conditions and high efficiencies can be upgraded into microbial protein, that is, protein‐rich biomass of microorganisms such as filamentous fungi, yeast, microalgae and bacteria (Areniello et al., 2022; Choi et al., 2022; Matassa et al., 2020). This approach utilizes what plants are very capable of doing: fixing CO_2_ into carbohydrates. This process, as depicted in Figure 2, combines the photosynthetic capacity of conventional carbohydrate‐producing plants with that of microbes, that is, the upgrading of the carbohydrates and mineral nutrients into valuable nutritious protein (Pikaar et al., 2017; Pikaar, Matassa, Bodirsky, et al., 2018; Pikaar, Matassa, Rabaey, et al., 2018).

**FIGURE 3:**
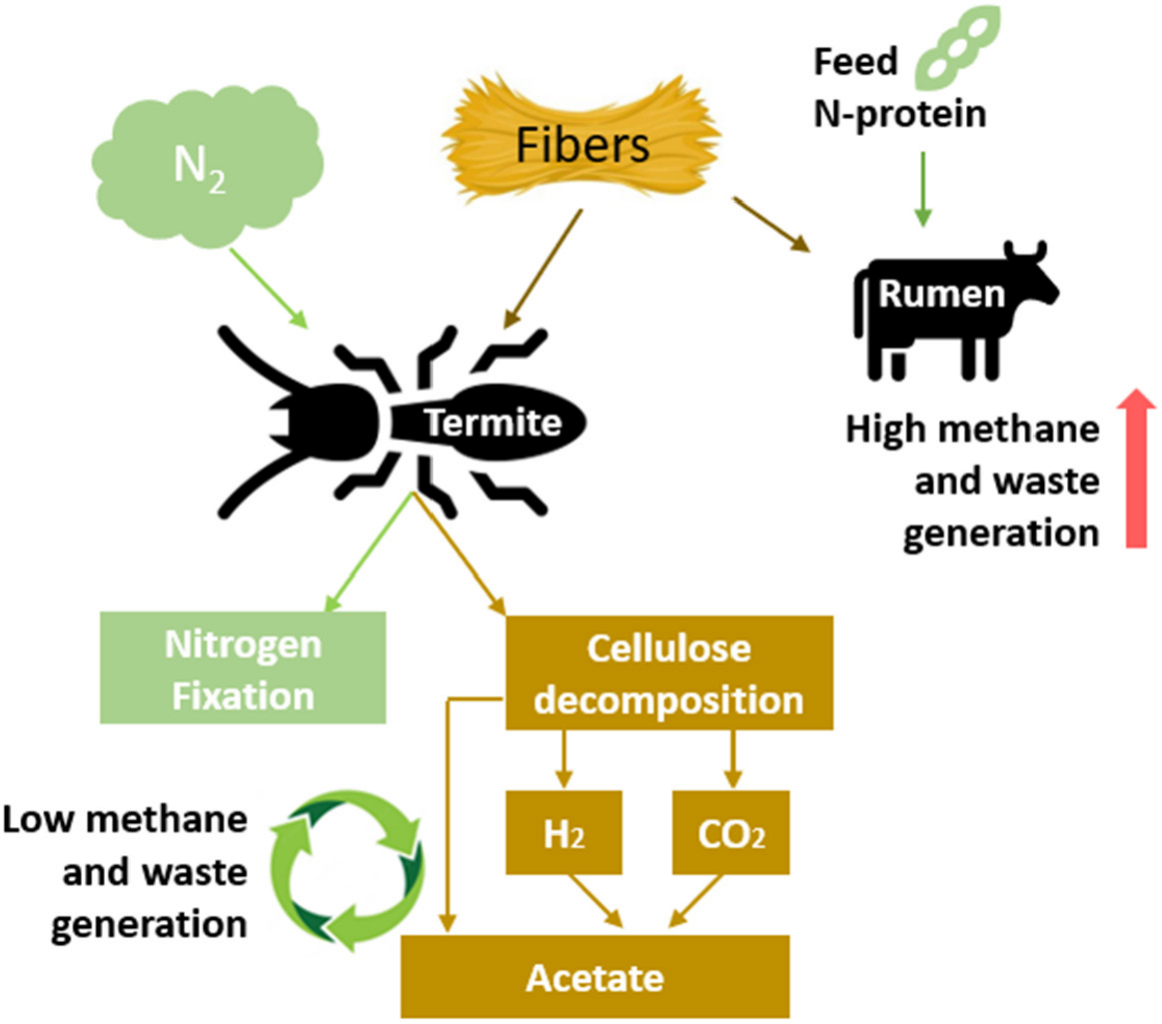
Qualitative comparison between the inefficient metabolic conversion of fibres and N‐protein through the rumen and the highly efficient metabolic fibre conversion and N‐fixation by means of termites (Upadhyaya et al., 2012). The termites are able to produce acetate as a source of both energy and carbon via cellulose decomposition and reductive acetogenesis thanks to their ability to convert about 74–99% (Brune, 2014) of wood's cellulose content. Also, in the termite gut, the production of reactive organic nitrogen thanks to nitrogen fixation makes termites independent of external reactive nitrogen supply (Ohkuma et al., 2015), while only a negligible amount of energy is wasted as methane as compared to ruminants (Conrad, 2009).

At present, this approach is possible. Globally, approximately 80 different microbial strains have been reported to produce food‐grade or feed‐grade proteins from a range of substrates (Banks et al., 2022). Notably, the production of yeast for feed or food purposes is well established (Matassa, Boon, et al., 2016; Matassa, Verstraete, et al., 2016). Also, the growth of fungal biomass to produce human food in the form of Quorn products is fully accepted and operational (Leger et al., 2021). It is generally considered that ruminants are highly suited to upgrade plant fibres (carbohydrates) to valuable proteins but, as highlighted in Figure 3, this route is quite problematic in terms of environmental sustainability, having a low feed conversion ratio and a high methane and waste generation (Beauchemin & McGinn, 2005; Loyon, 2018). Interestingly, insects such as wood‐consuming termites possess a more efficient overall pathway (Brune & Ohkuma, 2011). It remains to be seen to what extent biotechnological routes can be developed, which reach such ‘termite’ effectiveness in upgrading low‐value woody biomass. Moreover, in the termite gut, there is even fixation of dinitrogen from air into insect protein. The latter aspect of biological nitrogen fixation is outlined in more detail in Section 3, Boxes 1 and 2.

Box 2 **Nitrogen fixation by cyanobacteria**

Table 2 reports the mass of nitrogen that could be produced through N‐fixing autotrophic cyanobacteria cultivated in open raceway ponds. The fixation of nitrogen into cyanobacterial biomass could thus allow the production of about 4 tons of organic N per hectare per year. The latter productivity of reactive nitrogen under its organic form is in line with that estimated by Grizeau et al. (2016) for the photo‐production of soluble ammonium by heterocytous cyanobacteria. When compared to the productivity of reactive nitrogen in the soil system through N‐fixation in the root‐nodule‐rhizobia, ranging between 29 and 100 kg N per hectare per year (DeLuca et al., 2013; Reddy & Roger, 1985), 1 hectare of N‐fixing cyanobacterial culture could thus supply the same amount of reactive N provided by BNF to 40 to 138 hectares. Obviously, production prices in the range indicated in Table 2 make the process not yet competitive, yet the fact that the reactive N fixed through cyanobacteria could be applied under a liquid suspension could substantially lower the overall production cost, making such N‐fixation route more attractive.

**TABLE 2:** Productivity and production costs of N‐fixing cyanobacteria in open raceways ponds

Parameter	Value	Unit	Comments and references
Average N content of cyanobacteria	10%	% of N on DM	González López et al. (2010)
Areal productivity of open raceway ponds	20	g DM/m^2^·d	Moreno et al. (2003)
	40.000	kg DM/ha·y	Obtained by considering the production occurring for 200 days per year
	4000	kg N/ha·y	Obtained by considering the N content of cyanobacteria
Volumetric productivity of open raceway ponds	0.1	kg N/m^3^·d	Obtained by considering open ponds with 0.2 m water depth
Production cost of dry biomass produced in open raceway ponds	0.7–15	€/kg DM	Rafa et al. (2021)
	7–150	€/kg N	Obtained by considering the N content of cyanobacteria

### Establishing a circular nitrogen management system

Another line of action is to recover ‘used’ nitrogen. Used nitrogen could be present in solid, liquid or gas phases, for example, food waste, wastewater, etc. Taking N‐rich wastewater from food processing industry as an example, a wide range of used nitrogen sources (e.g. potato and soya‐processing wastewater, dairy wastewater, fish industry wastewater, fermentation wastewater) can be recovered via different pathways of which stripping of ammonia is the best known (Durkin et al., 2022; Lee & Stuckey, 2022).

In addition, the nitrogen consumed via the feed or food can be excreted via urine and faeces. Because in the fossil‐fuel‐based market economy, Haber–Bosch nitrogen was quite inexpensive, thus, the general trend was to ‘destroy’ the waste reactive nitrogen by conversion to N_2_. As indicated before, per person per day about 14 g of used nitrogen is excreted and is present in wastewater. In the wastewater treatment industry, a whole array of biotechnological processes have been designed to convert the nitrogen present in excrements in the form of proteins, urea and ammonium to dinitrogen so that it returns to the atmosphere. In principle, this was up to now a reasonable model because recovering ammonia nitrogen, for instance by means of stripping, costs some 2–3 Euros per kg N recovered, and therefore reclaiming used nitrogen was deemed too expensive (Matassa, Batstone, et al., 2015; Matassa, Boon, & Verstraete, 2015; Sigurnjak et al., 2019). Note that to achieve the removal of used nitrogen by converting it to dinitrogen, the processes involve the consumption of energy (i.e. fossil fuel) and unfortunately the process of nitrification and denitrification, by which the reactive nitrogen is turned to dinitrogen again, tends to lead to the emission of some nitrous oxide, which is a powerful greenhouse gas (see section 1). Taken together, the full arsenal of processes to destroy the ‘used’ nitrogen, as currently practiced in conventional water technology by means of processes such as nitrification, denitrification and anaerobic ammonium oxidation, can no longer be considered sustainable. They do not allow the re‐use of the precious asset of reactive nitrogen. However, faecal waste nitrogen is present in a very compromised matrix. Indeed, the faecal matter can contain various chemicals and microorganisms which in the context of hygiene and health have to be considered as undesirable (e.g. in terms of odour) or dangerous (infectious propagules). Hence, reactive nitrogen has first to be separated from the faecal matrix. The best‐developed process to achieve this is to strip the ammonia gas from the ‘waste’ matrix. Ammonia in gaseous form is subsequently trapped, most often in sulphuric acid, and thus becomes a fully clean resource (Matassa et al., 2020). Similarly, ammonia present in the gaseous emissions of stables (i.e., CAFOs), can be trapped by air washers in the form of ammonium sulphate. The problem thus far was that the regulator‐legislator had not established a framework for proper re‐use for recovered nitrogen, and overall the market demand was low due to its high costs as compared to conventional fertilizers (Matassa, Batstone, et al., 2015; Matassa, Boon, & Verstraete, 2015). Fortunately, the European Union has by the regulation 2019/1009 accepted the fact that ammonium sulphate, recovered by scrubbing of air coming from CAFOs, thermal driers, in acid, can attain by 16 July 2022 the status of regular fertilizer (European Union, 2019). Yet and very unfortunately, stripping of liquors (manure, digestates from various sludges, etc.) and subsequent scrubbing of these gases in acid is still not accepted by the EU regulator as a way to produce a ‘no‐longer‐ waste‐related’ quality fertilizer. Clearly, and it cannot be emphasized enough, just as the finding of Haber and Bosch changed the planet, the upgrading of used, and particularly faecal nitrogen to regulated fertilizer could become a major game changer in the overall governance of feed and food production and the sustainability of the planet. We need to fully develop this line of action. A circular economy is only feasible if the regulator focusses on the value of the recovered product, and this should be irrespective of the origin of the recovered product.

**FIGURE 4:**
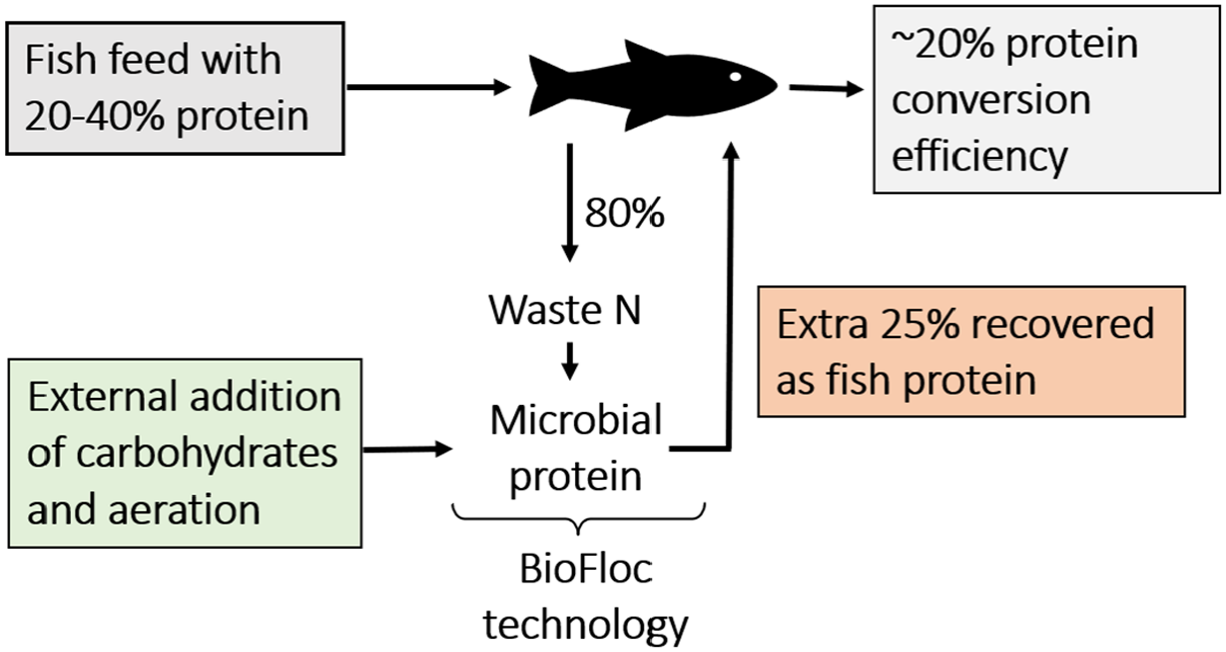
Working principle of the BioFloc technology (de Schryver et al., 2008).

A beautiful example indicating what can be achieved in terms of faecal nitrogen recovery and public acceptance is the practice of BioFloc Technology, as commonly applied in aquaculture (Figure 4). In this context, the external addition of a carbon and an energy source allows the microbial re‐assimilation of the waste ammonium nitrogen, excreted by the fish. The same fish will thus be able to gain additional energy and nitrogen from the protein‐rich microbial biomass synthesized through the recovered waste nitrogen. This quite revolutionary practice has allowed to more than double the protein conversion efficiency in aquaculture, and has been adopted all over the world. Currently, a relevant share of the fish food that we eat is being produced thanks to the BioFloc technology, proving how full public acceptance can be achieved also for such a kind of waste nitrogen recovery and upcycling approaches (de Schryver et al., 2008; de Schryver & Verstraete, 2009).

And why not use the stripped ammonia to make microbial proteins by using CO_2_? Actually, a very powerful approach consists of using the stripped ammonia to grow hydrogenotrophic bacteria (Matassa, Batstone, et al., 2015; Matassa, Boon, & Verstraete, 2015). This approach has been recently demonstrated and validated at the pilot scale within the Dutch national project ‘Power To Protein’, where the waste nitrogen recovered through stripping techniques from anaerobic sewage sludge digestate was subsequently upgraded into organic protein nitrogen in the form of high‐quality microbial proteins (Gavala et al., 2021). The latter were produced by fixing CO_2_ through the oxidation of hydrogen gas under aerobic conditions by means hydrogen‐oxidizing bacteria (Matassa, Boon, et al., 2016).

Similarly, recent studies demonstrated how the air stripping of nitrogen from anaerobic digestate could be combined in a clean and cost‐effective way with the aerobic fermentation of microbial protein from, thereby enabling the direct upcycling of waste nitrogen under the form of ammonia gas into protein (Matassa et al., 2022), or how combining the treatment of fertilizer plant wastewater and flue gases could lead to the production of protein‐rich microalgae (Molitor & Schnoor, 2022).

In the context of conventional sewage treatment plants which at present destroy most of the incoming nitrogen, the use of high‐rate algal ponds (HRAPs) particularly merits attention. The HRAPs use sunlight for direct nitrogen recovery through microalgae (−bacteria) production. Apparently, these systems already have fair removal efficiencies, their energy inputs are up to 50% lower than for conventional sewage treatment technologies (Ashraf et al., 2021), and they have the potential to be cost‐effective (Vulsteke et al., 2017). Provided further evolutions in engineering and modelling (Manhaeghe et al., 2020), improvement in biomass yields and lower production costs for these straightforward types of photobioreactors can be expected. In the same line of light‐driven recovery technologies, also the use of anaerobic purple bacteria in flat‐plate photobioreactors offers perspective for the upgrading of waste nitrogen and phosphorous into microbial biomass suitable for re‐use (Hülsen et al., 2022).

These positive experiences indicate the growing interest in combining nitrogen recovery with its valorization as high‐quality bioproducts such as microbial biomass.

Notwithstanding its potential advantages, at present, the use of microbial protein as feed or food is rather limited. The major barriers are twofold. First, there is the aspect of safety. When growing microorganisms in mass, one operates under conditions which can be favourable for potentially pathogenic species or species other than the one(s) that one aims for. Such mass production must be performed under well‐controlled conditions. Indeed, consumer fear of ‘contagion’ occurs, through lack of trust over the potential presence of organisms not considered to be safe. To overcome these safety issues, careful quality control needs to be provided, for instance generally applied in the food industry when conducting fermentations such as used when producing choucroute, wine, beer and dairy. Experience shows that excellent cheeses can be produced in an open environment from raw milk. Hence, there is no reason why microbial biomass, qualifying as top‐quality feeds and foods, could not be produced in bioreactors using spontaneous cultures. Yet, at present regulators consider that such new approaches, producing for instance microbial protein, require strict sterile conditions with only pure cultures of well‐known microbial species, generally already known and regarded as safe (de Vrieze et al., 2020). This very much slows down new developments in biotechnology; it has been argued that regulators should be more open‐minded on this issue and accept the wisdom of the past (Verstraete et al., 2022). The second hindrance relates to overall costs. Microbial biomass is normally high in moisture content (90% or more tightly bound water) and drying necessitates a cost of 9 units of water to be evaporated per unit of dry matter harvested, which is quite costly (Matassa et al., 2020). A line of development is to use liquors instead of powders, rich in microbial protein in our feed and food practices.

A final possible solution to valorize the reactive nitrogen content of sewage treatment plants, which might find an easier and more straightforward application due to the absence of links with the feed and food chain as well as with stringent regulations on chemicals such as the ‘Registration, Evaluation, Authorisation and Restriction of Chemicals’ (REACH) legislation (de Boer et al., 2018), may be the production of green hydrogen from ammonia and its subsequent use as renewable fuel (Grasham et al., 2020).

## FROM THE PAST, FORWARD TO THE FUTURE: THE RENAISSANCE OF ‘NITROGENASE’‐NITROGEN

At present, biological nitrogen fixation provides some 200 million tons of nitrogen to our planetary ecosystems. In terms of agriculture, biological nitrogen fixation plays a modest role; it provides some 25% of all nitrogen needed by crop plants (Islam, 2017). Indeed, chemical fertilizers are thus far sufficiently inexpensive and a technologically programmable method for crop production. Thus far, the demand for agricultural products produced in an eco‐friendly way is still quite restricted (in 2018 only some 1.3% of the Belgian agro‐area was strictly bio‐oriented) but the consumers who specifically choose environmental friendly food is growing (Timmermans & van Bellegem, 2018). Moreover, international policies such as the European Farm to Fork Strategy and the Biodiversity Strategy, as part of the European Green Deal, have set the ambition to establish a more sustainable agro‐ecology framework by (i) reducing by 20% the use of mineral fertilizers, (ii) decrease nutrient losses by 50%, (iii) bring back at least 10% of the current agricultural area under high‐diversity landscapes and (iv) employing 25% of the total agricultural area for organic farming (Montanarella & Panagos, 2021). The concept of a ‘protein shift’, particularly by using less chemical fertilizers, moving away from conventionally farmed protein and making more land available for other purposes, is gaining traction (Pikaar, Matassa, Bodirsky, et al., 2018; Pikaar, Matassa, Rabaey, et al., 2018). Before the invention of the Haber Bosch process to produce chemical fertilizers, the capture of atmospheric nitrogen relied on the activity of the microorganisms, and specifically on the catalytic activity of the nitrogenase enzyme present in these evolutionary ‘ancient’ forms (also called procaryotes) of life (Addo & dos Santos, 2020). The fact that higher forms of life do not possess the capacity to fix dinitrogen into reactive nitrogen is still an enigma, and this is often related to two special features of the nitrogenase. First, the nitrogenase can only operate in the strict absence of oxygen, hence it needs to be present in a special structure where oxygen diffusion is limited (Addo & dos Santos, 2020). The so‐called cyanobacteria (also called blue green algae) have solved this problem because they produce oxygen by photosynthesis and they have systems to ‘pack up’ the nitrogenase in heterocycsts where they fix nitrogen (Moreno et al., 2003). Also, some higher plants living in symbiosis with nitrogen‐fixing bacteria (rhizobium) have solved this by packing up the bacteria in nodules within their biomass (Vitousek et al., 2013). The Haber Bosch requires about 40 MJ under the form of fossil fuel to fix 1 kg of nitrogen (Box 1). Biological fixation requires ca. 30 kg of carbohydrates (or about 200 MJ) per kg nitrogen fixed (Box 1). In recent decades, 2.0 L of fossil fuel was much less expensive than 30 kg of sugar or starch. But times have changed and the fact that the carbohydrates can be acquired from a sustainable supply chain makes a new mindset possible. Consider for instance the fact that a single amino acid substitution in *Azotobacter vinelandii* allows to disrupt its control of the nitrogenase (Batista et al., 2021). The organism excretes ammonium outside the cell at a fair rate (~0.1 kg ammonium N per m^3^ per day) and at a fair yield (~100 units of carbohydrate ‐say 2 Euro of input per 1 kg of ammonia N produced) (Batista et al., 2021). In Box 1, the rates attained, calculated in terms of unit volume in which the microbial process takes place, suggest values from 0.02 to 0.75 kg N fixed per m^3^ bioreactor for the organotrophic bacteria. All this opens the perspective of engineering in‐reactor production of ammonia by using microbial nitrogenase driven by renewable carbon sources.

All together and independent of the enigma on the ‘loss of nitrogen‐fixing capacity in higher organisms’, the current status of the environment means that we must, by all means possible, decrease the use of fossil fuel and decrease the threats brought about by diffuse losses of reactive nitrogen species in the environment. This brings us to the question: has the time come to re‐examine the potentials of biological nitrogen fixation? The key feature of biological nitrogen fixation is that it relies on green renewable energy, and it is produced at the site of use where it is directly upgraded into protein. Notwithstanding the fact that the energy efficiency of the Haber Bosch efficiency is a factor 5–10 more efficient than that of biology, microorganisms have been responsible for the provision of reactive nitrogen of the planet for millions of years.

**FIGURE 5:**
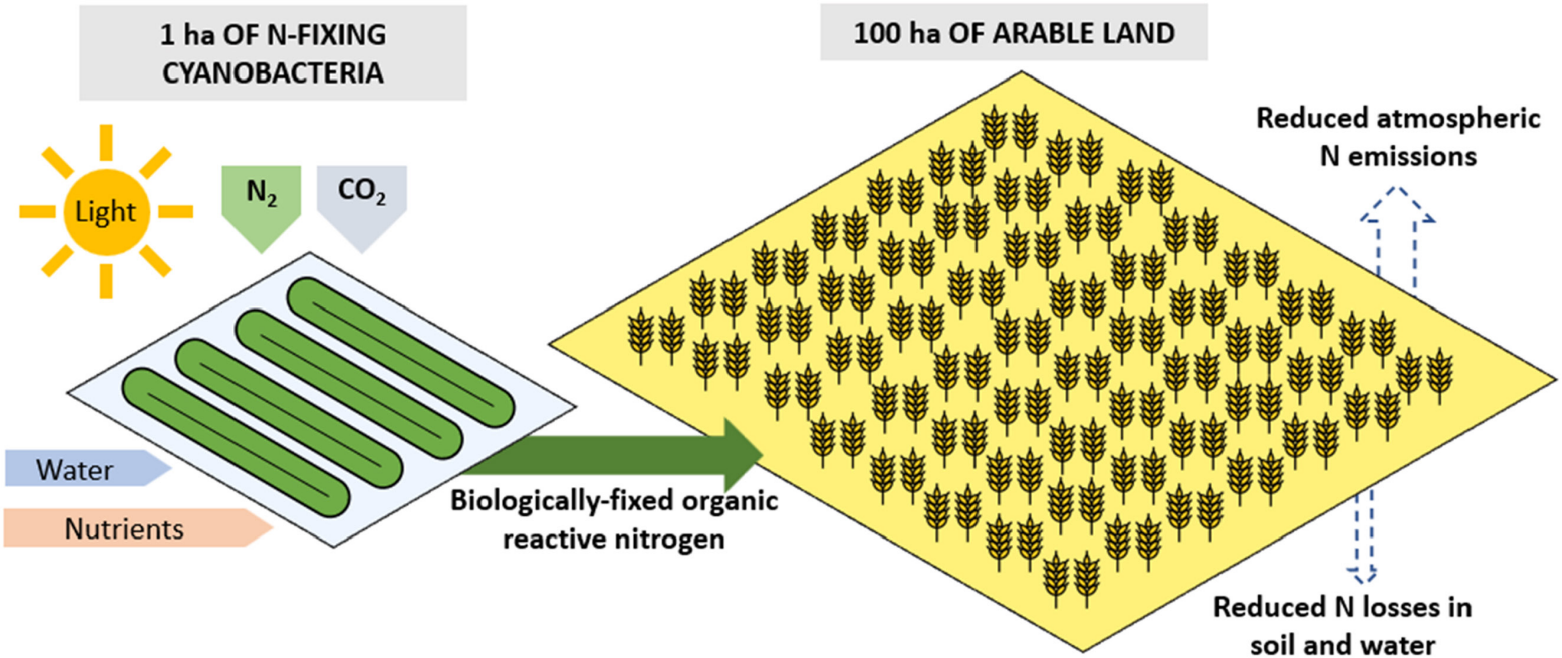
The concept of growing N‐fixing cyanobacteria to produce nitrogenase‐based organic nitrogen. The scheme highlights how the reactive nitrogen produced by 1 ha of N‐fixing cyanobacteria cultivation could supply enough nitrogenase‐based nitrogen to fertilize about 100 ha of agricultural land of high C/N crops that can be used in biorefinery as in Figure 2 (see Box 2).

Microorganisms can empower their nitrogenase by means of energy derived from organic matter. We call these as organotrophic nitrogen‐fixing bacteria. They consume carbohydrates produced by other plants by photosynthesis and although the efficiency of converting under the conditions of ambient temperatures is restricted, they are responsible for the fixation of massive amounts of nitrogen in the oceans and in terrestrial environments (Vitousek et al., 2013). Remarkable, Koh et al. (2022) have demonstrated that one can energize *Azotobacter* cultures by means of colloidal quantum dots to bring forward light‐driven ammonium production from di‐nitrogen gas. This brings this organotrophic route of nitrogen fixation in line with the already in nature existing direct route of light‐driven nitrogen fixation. Indeed, the autotrophic cyanobacteria can use sunlight and with that energy produce reactive nitrogen. Moreover, some of them can excrete excess ammonia fixed in their environment (Batista et al., 2021). Preliminary estimates from data at lab scale indicate that the amounts of ammonia nitrogen—upscaled from the mL scale in the lab to cubic meter reactor scale and per ha scale field reactor—can produce in the order of several thousands of kg of reactive N in the form of algal biomass nitrogen. In a first approximation, 1 ha dedicated to nitrogen fixation by cyanobacteria could be sufficient to supply some 100 ha of agricultural land in the surroundings, and all of this without the necessity of fossil fuel as the power source, but relying on the sun as an alternative (Box 2). Figure 5 depicts a concept of an N‐fixing algal farm producing organic reactive nitrogen to be used as an alternative fertilizer. The time has come to consider the design of cyanobacterial farming for environmentally friendly sustainable agriculture (Pathak et al., 2018). There is nothing that startling about this: the cyanobacterium *Spirulina* (*Arthrospira*) has been used by the Aztec population as food supplement for a long time (Pulz & Gross, 2004) and can contain up to 65% protein on dry weight (Wang et al., 2021).

## CONCLUDING REMARKS

In recent decades, fossil fuel was relatively inexpensive and so also was nitrogen produced through the Haber Bosch process. This has led to excessive application of nitrogen, which has become a threat to the sustainability of the planet. Remarkably, the current world situation in terms of fossil fuel prices has meant that Haber Bosch nitrogen strongly has risen in price.

Time has come to enter a post‐Haber–Bosch mindset and era. The need to use less fossil fuel‐based nitrogen fertilizer creates the necessity to use fertilizer more effectively and particularly to find legal contexts to valorize used, recovered nitrogen. Photobioreactor systems to upgrade used nitrogen to microbial biomass warrant further development. Quite special is the fact that fixing nitrogen from air by means of microbial nitrogenase systems offers potentials, both in terms of stoichiometry and rates, for future developments of these systems fitting both economic and ecological constraints. Most of all, the mindset of the consumer has changed in favour of fully bio‐based foods and thus a new era, in which ‘nitrogenase nitrogen’ plays a more dominant role than before, can be expected.

## CONFLICT OF INTEREST

The authors declare the absence of any potential competing or non‐financial interest.
